# Efficacy and Underpinnings of the Effigy in Wildlife Management

**DOI:** 10.3390/ani15172503

**Published:** 2025-08-25

**Authors:** Bryan M. Kluever, Mary J. Foley

**Affiliations:** 1 U.S. Department of Agriculture, Animal and Plant Health Inspection Service, Wildlife Services, National Wildlife Research Center, Florida Field Station, 2820 East University Avenue, Gainesville, FL 32641, USA; 2 U.S. Department of Agriculture, Animal and Plant Health Inspection Service, Wildlife Services, National Wildlife Research Center, 2820 East University Avenue, Fort Collins, CO 80521, USA

**Keywords:** antipredator, avian, deterrent, disgust, fear, neophobia

## Abstract

Displaying representations or actual individuals of the same or similar species can have a congregating or deterring effect on animals. An effigy, an animal or animal representation in a moribund state, is a tool aimed at deterring target animals in wildlife management settings. Despite being used as a deterrent for decades, neither a synthesis of effigies nor an exploration of the behavioral drivers tied to effigy effectiveness has occurred. We reviewed the effigy literature in terms of both efficacy as a deterrent and behavioral driver(s). We examined research on effigies encompassing 13 social avian species. Nine of seventeen (53%) investigations included experimentation and statistical validation, and of those, six of nine (66%) found effigies to be effective at deterring the focal species, though the temporal scale and spatial extents of the investigations were highly variable. We argue that central to an effigy’s effectiveness is the elicitation of fear in the target animal(s) and propose and discuss several potential drivers of fear derived from an effigy, namely antipredator behavior, neophobia, and disgust. Only a single study discussed behavioral drivers of effigy response. We conclude that effigies show promise as a wildlife deterrent for social avian species and have potential for uninvestigated species, and greater efforts should be made to incorporate robust study designs capable of strong inference and elucidation of behavioral drivers of an animal’s response to an effigy.

## Simple Summary

Animals can be attracted and deterred by members of the same or similar species and this behavior has aided wildlife management and conservation efforts. Along this vein, dead animals or artificial representations of dead animals, known as effigies, are presented to wildlife to make them flee from locations to reduce human–wildlife conflict. Despite being used by wildlife managers for decades, effigy effectiveness has never been reviewed. In addition, the behavioral drivers that might cause an effigy to be effective remain unexplored. Filling these knowledge gaps are needed to inform wildlife managers on which species effigies may be useful and to guide future effigy research. We reviewed the effigy literature and found 17 effigy studies involving 13 species, all birds. Slightly over half of the studies measured and tested for effigy effectiveness, and of these, two-thirds found effigies to be effective. Only one study linked effigy effectiveness to a behavioral driver, predation risk. We conclude that effigies show promise as a wildlife management tool but that many species remain unstudied and there is room for improvements in terms of study design and exploring why an effigy may or may not be effective.

## 1. Introduction

Examining and understanding how animals respond to biotic (e.g., predators, parasites, human disturbance) and abiotic (fire, chemical stressors) factors is a central tenet of animal behavior [1]. Whether a stimulus engenders fear and determining why said fear is manifested (or not) is an area investigated by basic and applied animal behavior practitioners alike [2,3]. For the latter, the study of fear in animals can have implications for both conservation and wildlife management. For example, eliciting a fear response in a species of conservation concern may be intentionally elicited to promote avoidance of ecological traps [1,2]. Within wildlife management, fear is often leveraged to mitigate human–wildlife conflict, with fleeing and/or avoidance by target individuals or populations being the desired outcome [4,5,6]. Equally germane to determining whether a human–wildlife conflict tool induces a measurable fear response (i.e., its efficacy) is identifying and understanding the underlying mechanism (s) driving a fear response and whether/how said mechanisms relate to existing theory (e.g., antipredator behavior theory). In fact, wildlife researchers have been called upon to better contemplate underlying mechanisms and test ecological/behavioral theory when developing and/or testing wildlife management practices [7].

**Figure 1:**
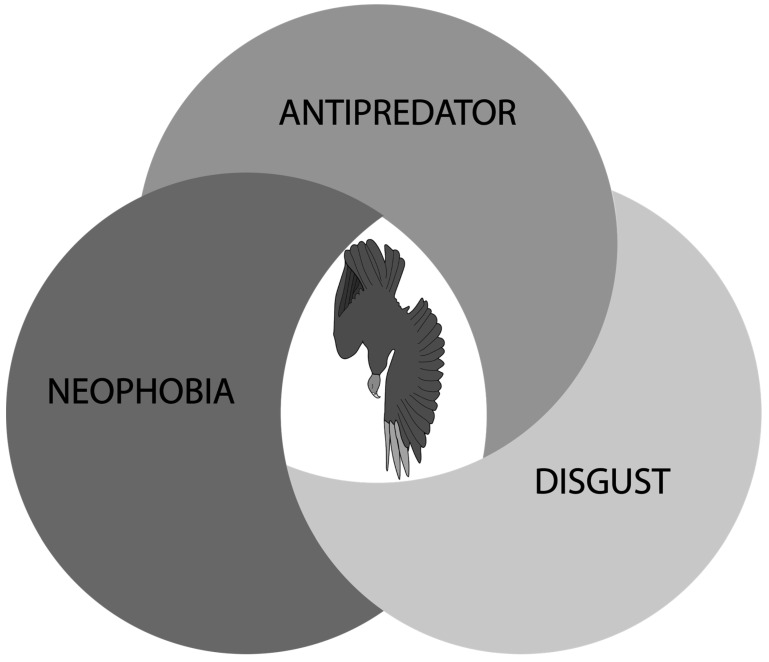
Visual framework for the potential behavioral attributions for when an effigy elicits a fear response. Multiple of these potential drivers of effigy fear response may contribute to effigy response simultaneously.

For millennia, humans have utilized artificial representations of humans and animals to alter the behavior of wildlife [8]. For example, actual animals or artificial representations of a conspecific or congener engaged in routine behaviors (e.g., wading, resting, feeding), often referred to as decoys, have been deployed to attract myriad species to a location for harvest or other activities (e.g., tag and release) [9,10]. However, at times converse to the intended consequences, decoys can have a repelling/dispersing effect, as was observed with wood pigeons (*Columba palambus*) [11]. Artificial representations of humans (i.e., scarecrows) and wildlife predators, at times referred to as effigies in the wildlife management field [12], have long been employed to promote fear in wildlife species, most notably involving birds and agriculture [13,14]. More recently, the term effigy has been used when describing an artificial or actual representation of a wildlife conspecific (or congener) in a moribund state, with both artificial (i.e., decoys) and real (i.e., carcasses) effigies being used as a tool to promote fear in a target species (Figure 1) [15,16]. Henceforth, we refer to this latter use of the term effigy.

A body of research exists on the fear response of animals in relation to predators and predation risk, including how fear-inducing antipredator behavior can affect individuals and populations [17,18,19,20]. More specifically, the human–wildlife conflict literature at times frames antipredator responses within a cost–benefit framework for understanding animal responses to tools (i.e., deterrents) intended to induce a fear response to reduce conflicts [3,5,21,22]. Similarly, other drivers of fear may contribute to tool effectiveness and can be viewed in terms of competing effects of the perceived costs and benefits of an adverse stimulus [23].

Novel stimuli can engender a suite of behavioral responses for animals that are inexperienced or lack a co-evolutionary history with the stimulus in question. Neophobia can occur in such cases, whereby a fear response is induced by a novel stimulus [24,25]. Multiple types of neophobia have been categorized, including fear of novel objects [26] and fear towards novel stimuli that are perceived as a possible predation threat (i.e., antipredator neophobia) [27]. Within the context of human–wildlife conflict and wildlife deterrents, both types of neophobia are most likely to be associated with a tool’s effectiveness. In terms of an effigy, neophobia may cause fear because the orientation of the deterrent is novel to target animals and/or this presentation of the effigy is perceived as being associated with a novel predation threat. For antipredator neophobia, a fear response can promote survival when facing unknown predators, whereas responses towards non-predators can result in both energetic and missed-opportunity costs [28].

Despite neophobia receiving increasing attention in biology and ecology, it is rarely explicitly contemplated as an underlying mechanism engendering a fear response via human–wildlife conflict tools, but see [29,30]. For example, in a contemporary review of human–wildlife conflict tools for invasive pest birds, neophobia was discussed only in terms of fear of traps (i.e., a novel object fear) as a challenge to trapping birds [31]. Many factors are thought to cause/contribute to neophobia, including exposure to high risk, small group size or social isolation, uncertainty about risk, and neural trauma [27].

In addition to predation risk and neophobia, disgust can also be a driver of fearful behavior. Disgust is an adaptive system hypothesized to have evolved to reduce the risk of becoming sick [32]. It is associated with behavioral, cognitive, and physiological responses that allow animals to avoid and/or get rid of parasites, pathogens, toxins, or contaminants. Disgust was formally conceptualized only recently in the animal behavior literature [28]. In terms of a behavioral response, disgust occurs when an animal modifies its behavior to avoid contact with a parasite, pathogen, toxin, or contaminant [33], all of which can negatively affect an animal’s fitness [34] and scale up to effects at the population level [35].

That avoidance behavior can result from disgust is compelling, as fear can also be elicited as result of antipredator behavior or neophobia, as discussed above. However, disgust, like neophobia, is rarely contemplated as a driver of fear resulting from exposure to a deterrent. For example, methyl anthranilate, a chemical repellent, has been tested for and employed as a tool for reducing human–wildlife conflict [36,37,38]. As a chemical repellent/irritant, the efficacy of methyl anthranilate is contingent upon an animal’s reaction following initial exposure [37]. That is, the disgust for the chemical repellent (and accompanying behavioral response) likely occurs by way of post-ingestive feedback [39] following initial or subsequent exposure. Clearly, there lies the potential for several alternative mechanistic underpinnings responsible for driving fear in an animal when exposed to deterrents or repellents.

To our knowledge, there has not been a literature review focusing on effigies in terms of efficacy or an exploration of the driver(s) of a fear response. A review of effigy efficacy can serve as a reference for wildlife practitioners contemplating the construction and deployment of effigies as a tool to reduce human–wildlife conflict by chronicling for what species the tool has shown effectiveness. A tandem examination of the literature in terms of the potential underling mechanisms/behavioral drivers that lead to fear in response to a conspecific effigy is also germane, as the aforementioned potential drivers of fear in wildlife—antipredator behavior, neophobia, and disgust—all have the potential to contribute to an effigy eliciting fear in the target species. The objectives of this paper were to 1) review and report on the existing wildlife literature on effigy efficacy, and 2) introduce and contemplate several mechanisms of realized or desired fear response from effigies, namely, predation, risk, neophobia, and disgust.

## 2. Materials and Methods

We conducted a literature search in October 2024. Search terms were developed for use in the common, peer-reviewed databases Web of Science and Scopus (Supplemental Table S1). We also searched the University of California-maintained Vertebrate Pest Conference archive because we surmised that a subset of the relevant literature on human–wildlife conflict and effigies would not be indexed (Supplemental Table S1). We suspected terms such as decoys and deterrent have been used interchangeably with effigy in the literature, so we included these terms in the search. Because we were interested in specifically reviewing the effigy literature, we excluded the terms models, scary/scary man, scarecrow, hawk-kite, kite balloon, and animal activated scarecrow, as we felt the inclusion of such terms would lead to more of a general review of visual-based wildlife deterrents, which has previously been undertaken [12,31].

From the output literature, we performed an initial screening of titles and abstracts and removed articles that clearly did not meet the intent of the literature review. For example, articles focusing on human or animal effigies found at human burial sites were removed. Following this screening, we determined whether the artificial animal representation described in each article was intended to portray a normal state (e.g., state feeding, wading, grooming) or moribund. Because we were specifically interested in reviewing the effigy literature within the context of human–wildlife conflict and fear, a full review of an article only continued if moribund state was the selected category. For these post-initial screening articles, we established eight criteria for further evaluation that we felt adequately captured the information needed to meet our objectives (Table 1).

## 3. Results

**Figure 2:**
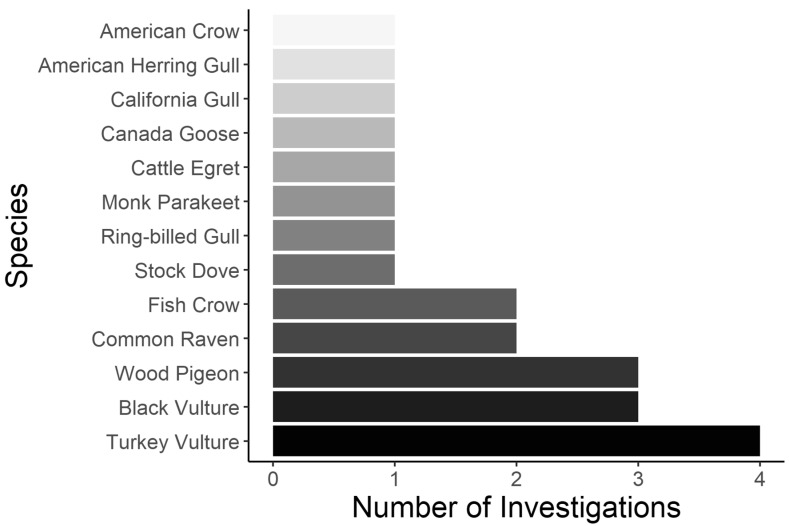
Frequency of investigations by species for a literature review on the use of effigies in wildlife management.

The literature search (Supplemental Table S1) yielded 119 total articles and 17 effigy investigations (Table 1). All effigy works were avian-focused and spanned 13 social species (Table 2). Multiple species were investigated more than once, with turkey vultures (*Cathartes aura*) being the most investigated (n = 4) (Figure 2). The number of effigies simultaneously deployed ranged from 1 to 100 (median = 1). Nine of the seventeen (53%) works included statistical quantification. Of these, six of nine (67%) provided evidence of effigies deterring the focal species. The temporal efficacy of effigies was highly variable, ranging from three hours to sixteen weeks (Table 2). Regarding effigy material, the number of investigations incorporating a carcass, taxidermy, and artificial effigies were twelve, seven, and seven, respectively. Regarding effigy type, most investigations (12 of 17) deployed conspecifics, with the other five incorporating both congener and conspecific effigies. Seven investigations (41%) attributed vision as the primary means of effigy detection while a sole study reported that olfaction contributed to effigy detection/reaction. Behavioral explanation in terms of effigy fear response (Table 1) was discussed in a single investigation (Table 2), with antipredator behavior being the purported driver of effigy-induced fear. All of the literature used the term “effigy”, minus one work, where the term “moribund decoy” was used. Ten of seventeen (58%) of the effigy investigations solely tested effigies, with the remaining works concurrently incorporating additional deterrents.

## 4. Discussion

### 4.1. Species Investigated

All effigy investigations were focused on social birds, with turkey vulture and black vulture (*Coragyps atratus*) being the most studied species (Figure 2). However, most of these vulture works lacked experimentation and statistical validation (Table 2), which means that caution is warranted when interpreting their findings and inference. Turkey and black vultures are known to utilize several roosts in a season, rotating amongst them independent of disturbance [55,56]. Effigy investigations that focus on one or few roosts may observe changes in vulture site attendance because of normal roost-switching behavior rather than effigy deployment, underscoring the need for ample replication across multiple populations of vultures and roost networks when investigating effigies. We speculate that social avian species are more frequently studied in effigy research compared to solitary species because the magnitude of human–wildlife conflict with social species is greater than with solitary species. When considering that only thirteen species were included in the review, there remains ample opportunity for effigy investigations on other social birds. For example, several species of crane regularly come into conflict with humans [57], and given their highly social nature, there is plausibility in testing hypotheses pertaining to the influence of effigies on their behavior.

Even though our review only detected avian-based effigy investigations, it is important to note that decoys for non-avian species (e.g., aquatic organisms, mammals) have been tested in the foraging behavior literature [58,59]; however, see [60]. In addition, necrophobic (the avoidance of dead or injured conspecific) behaviors have been documented within insects, aquatic organisms, and small mammals [61], though not within a human–wildlife conflict context. Further, marine mammal human–wildlife conflict has increased over time; despite this, it appears effigies have not been tested on this large group of animals [62]. Thus, we feel there is opportunity for effigy research in non-avian species, including crop-raiding mammals and invasive species

### 4.2. Efficacy

Nearly half of the effigy investigations lacked statistical quantification, being more anecdotal in nature or pilot studies, which does not allow for a robust test of efficacy. Avery et al. [43] claimed effigies aided in facilitating American crow (*Corvus brachyrhynchos*) and fish crow (*Corvus ossifragus*) roost abandonment, while Fellows and Patton [45] claimed effigy efficacy for cattle egrets (*Bubulcus ibis*). In a series of anecdotal site-specific investigations, Seamans et al. [53] claimed ring-billed gull (*Larus delawarensis*) and American herring gull (*Larus smithsonianus*) effigies were not effective if used alone but that they contributed to gull deterrence when used concomitantly with other deterrents. Ball [44] reported effigy effectiveness for turkey vultures, but without statistical validation. In another anecdotal investigation on monk parakeets (*Myiospitta monachus*) in Florida, Avery et al. [42] reported on the ineffectiveness of a taxidermy monk parakeet effigy. Caution is warranted when considering these anecdotal reports of effigy efficacy (or lack thereof), but these works can nonetheless inform future investigations of effigies that are designed to be more experimental.

Measures of effigy efficacy varied among investigations. Counts of birds were employed most often, but other metrics, such as nesting success [51], were also used. The appropriate measure for testing effigy efficacy will be largely contingent upon the type of human–wildlife conflict, and likely cannot be standardized across all future investigations. For example, long-term avoidance would be an appropriate measure to protect against damage to structures, such as communication towers, but for grain crops with a truncated damage window, short-term visitation would be more appropriate. Moreover, most grain crops are associated with very large fields, and in these settings, the effective use of effigies is likely infeasible given the sheer quantity of effigies that would likely be needed. With specialty crops associated with smaller fields, such as carrots, cucumbers, and leafy greens, effigy testing and implantation as a tool to reduce conflict is more likely than with grain crops. In sweetcorn, the period of deterrence is rarely more than two weeks [63]. Thus, a two-week temporal window could be appropriate for testing effigies for this crop, but for other grain crops with a larger damage window, such as sunflower, the temporal extent of data collection should be expanded.

Temporally, the maximum and minimum reported effigy effectiveness that included statistical validation was highly variable, ranging from three hours to sixteen weeks (Table 2). However, inferences from the report showing 16-week efficacy with California least terns (*Sterna antillarum browni*) and their seasonal nest success were confounded by additional management methods (e.g., pyrotechnics, horns) used alongside effigy deployment [51]. While Seamans [15] observed 12-week efficacy for a turkey vulture effigy, Avery [40,42] reporting effigy efficacy of up to five months for roosting black and/or turkey vultures, albeit without statistical evidence. Also, anecdotally, Hunter [46] reported that wood pigeon effigies reduced crop damage for up to five weeks, while Merrell [49] stated that effigies deterred common ravens from roost sites for several months. Temporal standardization of effigy investigations is likely not possible due to the wide range of species that can be tested and the specific associated human–wildlife conflicts (e.g., damage to property, damage to crops). However, we recommend studies incorporate, at minimum, a daily measure of effigy response across the temporal range associated with the human–wildlife conflict.

Regarding effigy material, only one study experimentally compared multiple types, revealing that, for wood pigeons, a crude artificial representation was outperformed by carcass effigies, though a more “realistic” molded plastic wood pigeon decoy conferred the same level of crop protection as carcasses [48]. In a simulated territorial intrusion investigation for European robins (*Erithacus rubecula*), Scriba and Goymann [64] found that birds reacted more aggressively to a taxidermy-based effigy compared to live decoys. Decoy type was also shown to influence attractiveness to wading birds [65]. Regardless of the type of effigy material, it is important to consider that moribund representations of animals may not align with social license; as a result, careful consideration should be taken when considering deploying effigies, especially on a large scale. Experimental comparisons of effigy materials appear to be a promising avenue of investigation. Contemporary examples of experimentally testing types of wildlife deterrent exist [29] and can be leveraged to inform study designs for future investigations.

Multiple additional factors have the potential to influence studies on effigies. For example, the spatial extent associated with an effigy test likely influences observed effectiveness. For example, when deploying black vulture effigies and handheld lasers, Avery et al. [42] anecdotally observed a vast reduction in black vultures at the roost site for nearly four months, but this did not affect black vulture use of nearby (4.2 to 22.3 km) livestock farms. Site familiarity/fidelity may also have an effect, as Forys et al. [16] suggested effigies were not effective for fish crows because the birds had already begun to depredate eggs within the roost prior to effigy deployment. Flight initiation distance may be a useful metric for measuring and accounting for site fidelity [29] prior to effigy deployment in future effigy tests; the distances that animals flee from an approaching human may correlate with effigy effectiveness.

Habitat type may also influence the effectiveness of effigies, and should be accounted for in future investigations. Testing effigies singularly rather than simultaneously with other deterrents is a requisite for determining inference and causality for this deterrent, but doing so is not without drawbacks, especially from an application standpoint. A dilemma occurs in human–wildlife conflict: it is widely affirmed that a combination of deterrents is likely necessary to reduce conflict to an appreciable level at a given site [66], but the efficacy of individual deterrents cannot be determined when deterrents are used in concert. A means of addressing this challenge is to incorporate a stepwise process, where effigies and other deterrents are first experimentally tested singularly, and then tested in combination. Without this step, interpretation and inference regarding effigies will continue to be hampered. Few of the effigy investigations incorporated control sites into the experimental design, most incorporating either an After or Before–After design [67]. These designs are not without merit (e.g., low to moderate relative cost) but they can produce unreliable estimates and incorrect inferences. As such, future effigy investigations would benefit by incorporating Before–After–Control–Impact or Control–Impact (BACI) designs [67], such as the work carried out by Peterson and Colwell [50] for common ravens (*Corvus corvax*). Once effigy efficacy is demonstrated for a particular species and wildlife management challenge, it should be incorporated into an integrated approach comprising several tools and community buy-in [66].

### 4.3. Behavioral Underpinnings

Effigy fear response was most often framed in the context of antipredator behavior rather than neophobia or disgust, but with the striking caveat that only one investigation discussed the behavioral drivers regarding effigies [47]. Vision was routinely framed as the driver of effigy detection (Table 2), and it seemed intuitive that when not explicitly mentioned, most investigations assumed that vision was the primary means of detecting an effigy. Through a series of experiments, Inglis and Isaacson [47] determined the white wing bands of the wood pigeon, which are only visible when the wings are outstretched, to be the visual cue responsible for the effectiveness of effigies for this species. However, Merrell [49] suspected that both sight and olfaction were responsible for effigy response in common ravens (*Corvus corax*).

We feel there is ample opportunity for future investigations into effigies to include an experimental testing of efficacy while simultaneously exploring the behavioral underpinnings of effigy response (or lack thereof). Behavioral assays with ample replication to allow for statistical validation should improve both the management and applications of effigies and our understanding of behavioral syndromes related to fear. For example, if individuals or populations react more fearfully to an effigy than a predator, this would provide evidence that disgust or neophobia likely contribute more to their reaction than antipredator behavior. Conversely, a more fearful reaction to an effigy when compared to novel objects provides evidence that predation risk, rather than neophobia, is the behavioral driver [47]. Further, neophobia tests [68] with multiple novel objects in addition to effigies should help determine if disgust or neophobia drive a fearful behavioral response. Disentangling antipredator and disgust behavior could potentially be achieved by exposing individuals or populations to carcass effigies in various levels of deterioration; a more fearful response to deteriorated effigies could provide evidence for disgust rather than predation risk. Leveraging captive animals (i.e., non-releasable raptors) may provide an opportunity to further our understanding of effigies, especially if assays are capable of being used across multiple facilities. Another potential area ripe for further research is determining if an effigy can be modified to enhance the desired fearful/repelling behavior, as was determined by expanding the wing band size for wood pigeon effigies [47].

In addition to the above, there is a rich amount of research on use of artificial representations of conspecifics or congeners to experimentally test behavioral hypotheses regarding competition [69,70,71], mate selection/attraction [72,73,74], and management actions such as translocation and colony recruitment [60,75,76,77,78]. This work, in addition to early effigy works that incorporated robust experimental designs [47,48], can be leveraged to guide future effigy studies capable of yielding behavioral insight

## 5. Conclusions

We provide a critical synthesis of the existing works on effigies. We conclude that effigies show promise as a wildlife deterrent for social avian species. Numerous social avian species that regularly conflict with humans (e.g., cranes, cormorants) remain untested, meriting future research. Effigy trials for non-avian species should also be considered in the future. Future investigations should consider behavioral drivers of effigy response, and when possible, incorporate experimental designs capable of testing various hypotheses related to antipredator behavior, neophobia, and disgust as potential behavioral drivers of effigies. This will be best achieved by framing future studies in a fear response framework, and we have provided numerous examples how this can be achieved. Greater efforts should be taken to avoid anecdotal effigy works by incorporating robust study designs capable of strong inference/statistical quantification. Doing so will better inform future wildlife management efforts.

## Figures and Tables

**Table 1:** Attributions and descriptions of evaluation criteria used to review the literature on the conspecific effigy, a tool aimed at inciting fear in and deterring wildlife.

Name	Type	Rationale for Inclusion
Focal Species	Qualitative	Identification of the species intended to respond to an effigy is needed to determine trends in effigy effectiveness or lack thereof.
Effigy Type	Categorical (conspecific, congener specific)	The response of a target species may be influenced by whether the effigy is a representation of the same or a similar species.
Effigy Material	Categorical (artificial, taxidermy, carcass)	The material comprising an effigy may influence the response of the target species.
Number of Effigies Deployed	Qualitative	The number of effigies simultaneously deployed at a site. For example, if study comprised three study sites with one effigy per site, the number = 1.
Effigy Effect Observed If Yes, Duration of Observed Effect	Categorical (Yes, No, NA) Continuous (maximum observed days)	Determining whether an effigy engendered a fear response is needed. Only validations of effectiveness associated with formal statistical quantification (non-anecdotal) were considered as a Yes or No. NA means the paper did not include statistical quantification.
Effigy Used Singularly	Binary (Yes, No)	In human–wildlife conflict settings, an integrated approach where multiple tools are deployed simultaneously occurs often. Replicating this in a research setting confounds inference in relation to each tools’ contribution to effectiveness. Determining whether effigies were used singularly or in concert with other deterrents is important to guide future investigations and to contemplate inference.
Physiological Attribution Considered?	Binary (Yes, No) If Yes, Qualitative	Though our primary focus beyond effigy efficacy was exploring potential behavioral driver of an effigy response, an exploration/consideration of the physiological means of effigy detection should be tallied.
Behavioral Explanation Contemplated?	Binary (Yes, No) If Yes, Qualitative	Several drivers of fear may influence an animal’s response to an effigy. Contemplation of the causality of fear in relation to an effigy is necessary for better understanding the effigy as a wildlife deterrent.

**Table 2:** Summary of the literature review findings on use of animal effigies. See Supplemental Table S1 for further details on the literature review process beyond the description provided in the Methods Section. See Table 1 for justification/rationale for inclusion and a full explanation of each field/column. An “NA” represents a lack of experimentation and statistical validation. In the investigation noted by “*”, authors tested for the effectiveness of wings only in addition to full carcasses.

Citation	Focal Species	Effigy Type	Effigy Material	Number of Effigies Deployed	Effigy Effect Observed?	Effigy Used Singularly?	Physiological Attribution Considered?	Behavioral Explanation Contemplated?
Avery et al. 2002a [40]	Black Vulture Turkey Vulture	Both	Artificial Carcass Taxidermy	1	Yes 9 days	Yes	Yes Visual	No
Avery et al. 2002b [41]	Monk Parakeet	Conspecific	Taxidermy	1	NA	Yes	No	No
Avery et al. 2006 [42]	Black Vulture	Conspecific	Taxidermy Carcass	4	No	No	No	No
Avery et al. 2008 [43]	American Crow Fish Crow	Both	Artificial Carcass Taxidermy	1 to 5	NA	No	Yes Visual	No
Ball 2009 [44]	Turkey Vulture	Conspecific	Taxidermy	1 to 2	NA	Yes	No	No
Fellows and Patton 1988 [45]	Cattle Egret	Conspecific	Carcass	Unknown	NA	No	No	No
Forys et al. 2015 [16]	Fish Crow	Conspecific	Artificial	6	No	Yes	No	No
Hunter 1974 [46]	Wood Pigeon	Conspecific	Artificial Carcass	100	NA	Yes	No	No
Inglis and Isaacson 1984 [47]	Wood Pigeon Stock dove	Both	Carcass*	10	Yes 3 h	Yes	Yes Visual	Yes
Inglis and Isaacson 1987 [48]	Wood pigeon	Conspecific	Artificial Carcass*	9, 20	Yes 9 weeks	Yes	Yes Visual	No
Merrell 2012 [49]	Common Raven	Conspecific	Artificial Carcass	2 to 3	NA	Yes	Yes Visual Olfaction	No
Peterson and Colwell 2014 [50]	Common Raven	Conspecific	Carcass	1	Yes 3 days	No	No	No
Riensche et al. 2012 [51]	California Gull	Conspecific	Carcass	Unknown	Yes 16 weeks	No	No	No
Seamans 2004 [15]	Turkey Vulture	Conspecific	Taxidermy	1	Yes 12 weeks	Yes	Yes Visual	No
Seamans and Bernhardt 2004 [52]	Canada Goose	Conspecific	Artificial	2	No	Yes	No	No
Seamans et al. 2007 [53]	American Herring Gull Ring-billed Gull	Both	Carcass	4 to 8	NA	No	No	No
Tillman et al. 2002 [54]	Black Vulture Turkey Vulture	Both	Artificial Carcass Taxidermy	1 to 4	NA	Yes	Yes Visual	No

## Data Availability

Data is available upon request by contacting corresponding author.

## Supplementary Materials

The following supporting information can be downloaded at: https://www.mdpi.com/article/10.3390/ani15172503/s1, Table S1: Literature Search that led full to full screening of 119 articles, 17 of which were effigy investigations tied to wildlife management.
