# Supplementary material for: Efficacy and Underpinnings of the Effigy in Wildlife Management
Source: Animals (Basel). 2025 Aug 25;15(17):2503. doi: 10.3390/ani15172503 (PMC12427257; doi:10.3390/ani15172503)
Supplement: Supplementary file 1 [file animals-15-02503-s001.zip › animals-3781170-supplementary.pdf]

Supplemental Table S1. Literature search that led full to a full screening of 119 articles, 17 of which were effigy investigations tied to wildlife management.

**Database**	**Search Terms**	**Results**
Web of Science	TS=((effigy OR effigies OR decoy OR decoys) AND ((deter* OR conflict or behavior* OR damag* OR respons* OR scar*) AND (wildlife OR bird OR birds OR mammal OR mammals OR reptile OR reptiles OR animal)))	439, sorted to 63
Scopus	TITLE-ABS-KEY ((effigy OR effigies OR decoy OR decoys) AND ((deter* OR conflict OR behavior* OR damag* OR respons* OR scar*) AND (wildlife OR bird OR birds OR mammal OR mammals OR reptile OR reptiles OR animal)))	2079, sorted to 38
Vertebrate Pest Conference<br><br>eScholarship, Open Access Publications from the University of California	decoy or effigy	43, sorted to 18
